# Supplementary figures and images for: The modified 30-second chair stand test (m-30s-CST) is more sensitive than handgrip strength in detecting muscle strength changes and predicting physical performance in hospitalized geriatric patients
Source: PLoS One. 2026 Mar 16;21(3):e0331155. doi: 10.1371/journal.pone.0331155 (PMC12991214; doi:10.1371/journal.pone.0331155)

**S1 Figure. Flowchart of patient inclusion.**

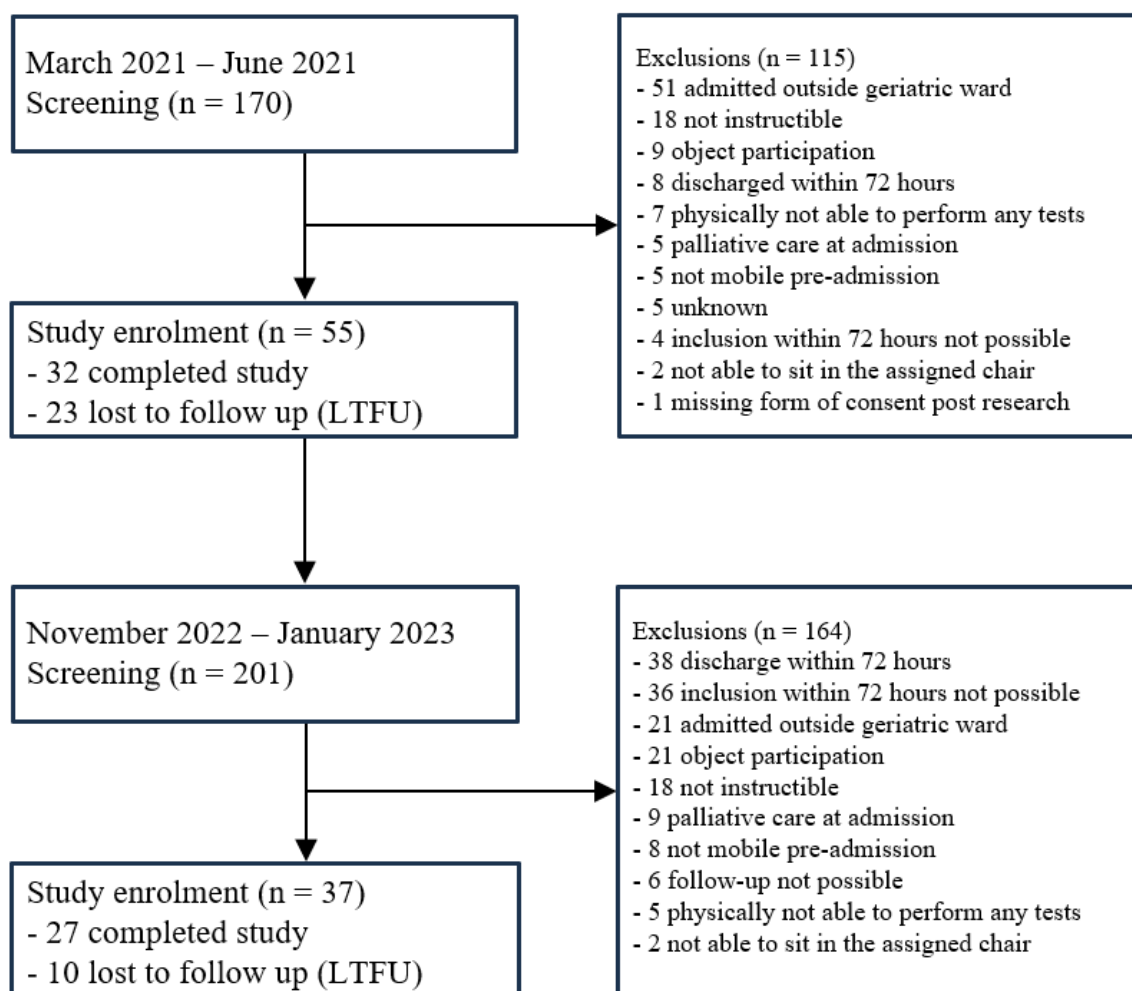

Supplement: S1 Fig — (PDF) [file pone.0331155.s001.pdf]
