# Supplementary material for: The modified 30-second chair stand test (m-30s-CST) is more sensitive than handgrip strength in detecting muscle strength changes and predicting physical performance in hospitalized geriatric patients
Source: PLoS One. 2026 Mar 16;21(3):e0331155. doi: 10.1371/journal.pone.0331155 (PMC12991214; doi:10.1371/journal.pone.0331155)
Supplement: S1 Table — * p < 0.05 indicates statistically significant difference. (PDF) [file pone.0331155.s002.pdf]

S2 Table. Impact of patient characteristics on the patients who improved compared to the patients who remained stable or worsened on the SPPB in hospitalized geriatric patients (N=76)

	n	SPPB improved	n	SPPB stable or worse	Difference *P*
Age	41	84.2 ± 5.7	35	82.7 ± 7.4	0.162
GFI	41	6.7 ± 2.9	35	5.2 ± 2.9	0.017*
CCI	41	6.1 ± 2.0	35	6.2± 2.0	0.390
SNAQ	41	1.9 ± 1.8	35	1.5 ± 1.6	0.163
Hospital stay	41	9.7 ± 10.5	35	10.3 ± 11.4	0.418

* $p < 0.05$ indicates statistically significant difference.
