# Supplementary material for: The modified 30-second chair stand test (m-30s-CST) is more sensitive than handgrip strength in detecting muscle strength changes and predicting physical performance in hospitalized geriatric patients
Source: PLoS One. 2026 Mar 16;21(3):e0331155. doi: 10.1371/journal.pone.0331155 (PMC12991214; doi:10.1371/journal.pone.0331155)
Supplement: S3 Table — * p < 0.05 indicates statistically significant difference. (PDF) [file pone.0331155.s004.pdf]

S3 Table: Muscle strength, physical function, age, comorbidity, frailty, nutrition and BMI versus 2-year survival in acutely hospitalized geriatric patients (n=92).

**2-year mortality predictor**	**alive**		**deceased**		**difference**
	n	mean	n	mean	*P*
**m-30s-CST**	43	5.2 ± 4.4	49	3.5 ± 3.0	0.014*
**HGS Jamar**	43	17.8 ±10.2	49	15.3 ± 7.7	0.094
**SPPB**	43	3.1 ± 2.6	49	2.6 ± 2.3	0.178
**ADL-Barthel Index**	43	12.7± 4.2	49	12.1 ± 4.5	0.257
**CCI**	43	6.3 ± 1.9	49	6.3 ± 2.1	0.496
**Age**	43	83.9 ± 6.6	49	83.9 ± 6.2	0.490
**GFI**	43	6.2 ± 3.1	49	6.1 ± 2.8	0.424
**SNAQ**	43	1.7 ± 1.8	49	1.7 ± 1.7	0.456
**BMI**	43	24.2 ± 3.3	49	25.2 ± 3.7	0.094

* $p < 0.05$ indicates statistically significant difference.
