# Supplementary material for: The modified 30-second chair stand test (m-30s-CST) is more sensitive than handgrip strength in detecting muscle strength changes and predicting physical performance in hospitalized geriatric patients
Source: PLoS One. 2026 Mar 16;21(3):e0331155. doi: 10.1371/journal.pone.0331155 (PMC12991214; doi:10.1371/journal.pone.0331155)
Supplement: S1 Data — (DOCX) [file pone.0331155.s005.docx]

**Minimal dataset.docx.**

Kaplan-Meier

| **Case Processing Summary** | | | | |
| --- | --- | --- | --- | --- |
| M30CST_CAT6 | Total N | N of Events | Censored | |
|  |  |  | N | Percent |
| goed: >5 | 32 | 11 | 21 | 65,6% |
| slecht: <6 | 60 | 38 | 22 | 36,7% |
| Overall | 92 | 49 | 43 | 46,7% |

| **Survival Table** | | | | | | | |
| --- | --- | --- | --- | --- | --- | --- | --- |
| M30CST_CAT6 | | Time | Status | Cumulative Proportion Surviving at the Time | | N of Cumulative Events | N of Remaining Cases |
|  |  |  |  | Estimate | Std. Error |  |  |
| goed: >5 | 1 | 19,000 | overleden | ,969 | ,031 | 1 | 31 |
|  | 2 | 21,000 | overleden | ,938 | ,043 | 2 | 30 |
|  | 3 | 37,000 | overleden | ,906 | ,052 | 3 | 29 |
|  | 4 | 41,000 | overleden | ,875 | ,058 | 4 | 28 |
|  | 5 | 57,000 | overleden | ,844 | ,064 | 5 | 27 |
|  | 6 | 103,000 | overleden | ,813 | ,069 | 6 | 26 |
|  | 7 | 275,000 | overleden | ,781 | ,073 | 7 | 25 |
|  | 8 | 387,000 | overleden | ,750 | ,077 | 8 | 24 |
|  | 9 | 594,000 | overleden | ,719 | ,079 | 9 | 23 |
|  | 10 | 607,000 | overleden | ,688 | ,082 | 10 | 22 |
|  | 11 | 608,000 | overleden | ,656 | ,084 | 11 | 21 |
|  | 12 | 730,000 | levend | . | . | 11 | 20 |
|  | 13 | 730,000 | levend | . | . | 11 | 19 |
|  | 14 | 730,000 | levend | . | . | 11 | 18 |
|  | 15 | 730,000 | levend | . | . | 11 | 17 |
|  | 16 | 730,000 | levend | . | . | 11 | 16 |
|  | 17 | 730,000 | levend | . | . | 11 | 15 |
|  | 18 | 730,000 | levend | . | . | 11 | 14 |
|  | 19 | 730,000 | levend | . | . | 11 | 13 |
|  | 20 | 730,000 | levend | . | . | 11 | 12 |
|  | 21 | 730,000 | levend | . | . | 11 | 11 |
|  | 22 | 730,000 | levend | . | . | 11 | 10 |
|  | 23 | 730,000 | levend | . | . | 11 | 9 |
|  | 24 | 730,000 | levend | . | . | 11 | 8 |
|  | 25 | 730,000 | levend | . | . | 11 | 7 |
|  | 26 | 730,000 | levend | . | . | 11 | 6 |
|  | 27 | 730,000 | levend | . | . | 11 | 5 |
|  | 28 | 730,000 | levend | . | . | 11 | 4 |
|  | 29 | 730,000 | levend | . | . | 11 | 3 |
|  | 30 | 730,000 | levend | . | . | 11 | 2 |
|  | 31 | 730,000 | levend | . | . | 11 | 1 |
|  | 32 | 739,000 | levend | . | . | 11 | 0 |
| slecht: <6 | 1 | 2,000 | overleden | ,983 | ,017 | 1 | 59 |
|  | 2 | 9,000 | overleden | . | . | 2 | 58 |
|  | 3 | 9,000 | overleden | ,950 | ,028 | 3 | 57 |
|  | 4 | 14,000 | overleden | . | . | 4 | 56 |
|  | 5 | 14,000 | overleden | ,917 | ,036 | 5 | 55 |
|  | 6 | 15,000 | overleden | ,900 | ,039 | 6 | 54 |
|  | 7 | 20,000 | overleden | ,883 | ,041 | 7 | 53 |
|  | 8 | 23,000 | overleden | ,867 | ,044 | 8 | 52 |
|  | 9 | 31,000 | overleden | ,850 | ,046 | 9 | 51 |
|  | 10 | 36,000 | overleden | . | . | 10 | 50 |
|  | 11 | 36,000 | overleden | ,817 | ,050 | 11 | 49 |
|  | 12 | 41,000 | overleden | ,800 | ,052 | 12 | 48 |
|  | 13 | 44,000 | overleden | ,783 | ,053 | 13 | 47 |
|  | 14 | 45,000 | overleden | ,767 | ,055 | 14 | 46 |
|  | 15 | 58,000 | overleden | ,750 | ,056 | 15 | 45 |
|  | 16 | 73,000 | overleden | ,733 | ,057 | 16 | 44 |
|  | 17 | 77,000 | overleden | . | . | 17 | 43 |
|  | 18 | 77,000 | overleden | ,700 | ,059 | 18 | 42 |
|  | 19 | 83,000 | overleden | ,683 | ,060 | 19 | 41 |
|  | 20 | 85,000 | overleden | ,667 | ,061 | 20 | 40 |
|  | 21 | 86,000 | overleden | ,650 | ,062 | 21 | 39 |
|  | 22 | 93,000 | overleden | ,633 | ,062 | 22 | 38 |
|  | 23 | 103,000 | overleden | ,617 | ,063 | 23 | 37 |
|  | 24 | 115,000 | overleden | ,600 | ,063 | 24 | 36 |
|  | 25 | 141,000 | overleden | ,583 | ,064 | 25 | 35 |
|  | 26 | 167,000 | overleden | ,567 | ,064 | 26 | 34 |
|  | 27 | 251,000 | overleden | ,550 | ,064 | 27 | 33 |
|  | 28 | 334,000 | overleden | ,533 | ,064 | 28 | 32 |
|  | 29 | 353,000 | overleden | ,517 | ,065 | 29 | 31 |
|  | 30 | 405,000 | overleden | ,500 | ,065 | 30 | 30 |
|  | 31 | 419,000 | overleden | ,483 | ,065 | 31 | 29 |
|  | 32 | 427,000 | overleden | ,467 | ,064 | 32 | 28 |
|  | 33 | 501,000 | overleden | ,450 | ,064 | 33 | 27 |
|  | 34 | 580,000 | overleden | ,433 | ,064 | 34 | 26 |
|  | 35 | 587,000 | overleden | ,417 | ,064 | 35 | 25 |
|  | 36 | 655,000 | overleden | ,400 | ,063 | 36 | 24 |
|  | 37 | 657,000 | overleden | ,383 | ,063 | 37 | 23 |
|  | 38 | 675,000 | overleden | ,367 | ,062 | 38 | 22 |
|  | 39 | 730,000 | levend | . | . | 38 | 21 |
|  | 40 | 730,000 | levend | . | . | 38 | 20 |
|  | 41 | 730,000 | levend | . | . | 38 | 19 |
|  | 42 | 730,000 | levend | . | . | 38 | 18 |
|  | 43 | 730,000 | levend | . | . | 38 | 17 |
|  | 44 | 730,000 | levend | . | . | 38 | 16 |
|  | 45 | 730,000 | levend | . | . | 38 | 15 |
|  | 46 | 730,000 | levend | . | . | 38 | 14 |
|  | 47 | 730,000 | levend | . | . | 38 | 13 |
|  | 48 | 730,000 | levend | . | . | 38 | 12 |
|  | 49 | 730,000 | levend | . | . | 38 | 11 |
|  | 50 | 730,000 | levend | . | . | 38 | 10 |
|  | 51 | 730,000 | levend | . | . | 38 | 9 |
|  | 52 | 730,000 | levend | . | . | 38 | 8 |
|  | 53 | 730,000 | levend | . | . | 38 | 7 |
|  | 54 | 730,000 | levend | . | . | 38 | 6 |
|  | 55 | 730,000 | levend | . | . | 38 | 5 |
|  | 56 | 730,000 | levend | . | . | 38 | 4 |
|  | 57 | 730,000 | levend | . | . | 38 | 3 |
|  | 58 | 730,000 | levend | . | . | 38 | 2 |
|  | 59 | 730,000 | levend | . | . | 38 | 1 |
|  | 60 | 730,000 | levend | . | . | 38 | 0 |

| **Means and Medians for Survival Time** | | | | | | |
| --- | --- | --- | --- | --- | --- | --- |
| M30CST_CAT6 | Mean^a^ | | | | Median | |
|  | Estimate | Std. Error | 95% Confidence Interval | | Estimate | Std. Error |
|  |  |  | Lower Bound | Upper Bound |  |  |
| goed: >5 | 570,875 | 48,148 | 476,505 | 665,245 | . | . |
| slecht: <6 | 390,017 | 40,137 | 311,348 | 468,686 | 405,000 | 161,374 |
| Overall | 455,076 | 32,549 | 391,280 | 518,872 | 608,000 | . |

| **Means and Medians for Survival Time** | | | | |  |  |  |  |
| --- | --- | --- | --- | --- | --- | --- | --- | --- |
| M30CST_CAT6 | Median^a^ | | | |  |  |  |  |
|  | 95% Confidence Interval | | | |  |  |  |  |
|  | Lower Bound | | Upper Bound | |  |  |  |  |
| goed: >5 | . | | . | |  |  |  |  |
| slecht: <6 | 88,706 | | 721,294 | |  |  |  |  |
| Overall | . | | . | |  |  |  |  |
|  |  |  | |  | |  |  |  |

| a. Estimation is limited to the largest survival time if it is censored. |
| --- |

| **Overall Comparisons** | | | |
| --- | --- | --- | --- |
|  | Chi-Square | df | Sig. |
| Log Rank (Mantel-Cox) | 6,806 | 1 | ,009 |
| Breslow (Generalized Wilcoxon) | 6,521 | 1 | ,011 |
| Tarone-Ware | 6,733 | 1 | ,009 |
| Test of equality of survival distributions for the different levels of M30CST_CAT6. | | | |

**ROC Curve**

| **Case Processing Summary** | |
| --- | --- |
| Overlijden < 2 jaar | Valid N (listwise) |
| Positive^a^ | 43 |
| Negative | 49 |
| Larger values of the test result variable(s) indicate stronger evidence for a positive actual state. | |
| a. The positive actual state is levend. | |

| **Area Under the Curve** | | | | | |
| --- | --- | --- | --- | --- | --- |
| Test Result Variable(s) | Area | Std. Error^a^ | Asymptotic Sig.^b^ | Asymptotic 95% Confidence Interval | |
|  |  |  |  | Lower Bound | Upper Bound |
| m30s_CST_t0 | ,609 | ,062 | ,071 | ,488 | ,731 |
| HGS_Jamar_t0 | ,573 | ,060 | ,228 | ,455 | ,691 |
| The test result variable(s): m30s_CST_t0, HGS_Jamar_t0 has at least one tie between the positive actual state group and the negative actual state group. Statistics may be biased. | | | | | |
| a. Under the nonparametric assumption | | | | | |
| b. Null hypothesis: true area = 0.5 | | | | | |

| **Coordinates of the Curve** | | | |
| --- | --- | --- | --- |
| Test Result Variable(s) | Positive if Greater Than or Equal To^a^ | Sensitivity | 1 - Specificity |
| m30s_CST_t0 | -1,00 | 1,000 | 1,000 |
|  | ,50 | ,744 | ,776 |
|  | 1,50 | ,698 | ,755 |
|  | 2,50 | ,628 | ,612 |
|  | 3,50 | ,628 | ,367 |
|  | 4,50 | ,558 | ,306 |
|  | 5,50 | ,488 | ,224 |
|  | 6,50 | ,419 | ,122 |
|  | 7,50 | ,279 | ,102 |
|  | 8,50 | ,233 | ,102 |
|  | 9,50 | ,186 | ,041 |
|  | 10,50 | ,163 | ,020 |
|  | 11,50 | ,116 | ,020 |
|  | 12,50 | ,047 | ,000 |
|  | 14,00 | ,023 | ,000 |
|  | 16,00 | ,000 | ,000 |
| HGS_Jamar_t0 | -1,00 | 1,000 | 1,000 |
|  | 1,00 | ,977 | 1,000 |
|  | 2,50 | ,977 | ,980 |
|  | 4,50 | ,977 | ,959 |
|  | 6,50 | ,907 | ,878 |
|  | 7,50 | ,884 | ,857 |
|  | 9,00 | ,837 | ,816 |
|  | 10,50 | ,744 | ,714 |
|  | 11,50 | ,744 | ,633 |
|  | 12,50 | ,651 | ,551 |
|  | 13,50 | ,628 | ,531 |
|  | 15,00 | ,581 | ,449 |
|  | 16,50 | ,535 | ,347 |
|  | 17,50 | ,465 | ,327 |
|  | 19,00 | ,419 | ,245 |
|  | 20,50 | ,372 | ,224 |
|  | 21,50 | ,326 | ,224 |
|  | 22,50 | ,279 | ,204 |
|  | 23,50 | ,256 | ,184 |
|  | 25,00 | ,163 | ,143 |
|  | 26,50 | ,116 | ,143 |
|  | 27,50 | ,116 | ,102 |
|  | 28,50 | ,093 | ,082 |
|  | 29,50 | ,070 | ,061 |
|  | 30,50 | ,047 | ,041 |
|  | 31,50 | ,023 | ,041 |
|  | 32,50 | ,023 | ,020 |
|  | 47,50 | ,023 | ,000 |
|  | 63,00 | ,000 | ,000 |
| The test result variable(s): m30s_CST_t0, HGS_Jamar_t0 has at least one tie between the positive actual state group and the negative actual state group. | | | |
| a. The smallest cutoff value is the minimum observed test value minus 1, and the largest cutoff value is the maximum observed test value plus 1. All the other cutoff values are the averages of two consecutive ordered observed test values. | | | |

**General Linear Model**

[DataSet1] C:\Users\151390\OneDrive - Zuyderland\walther\onderzoek\CST\prognose m-30s-CST\Plos One\kracht prognostische waarde jan 2025.sav

| **Within-Subjects Factors** | |
| --- | --- |
| Measure: MEASURE_1 | |
| factor1 | Dependent Variable |
| 1 | m30s_CST_t0 |
| 2 | m30s_CST_tdbd |

| **Between-Subjects Factors** | | | |
| --- | --- | --- | --- |
|  | | Value Label | N |
| Barthel beter of stabiel-slechter | 1 | verbetering | 43 |
|  | 2 | stabiel of verslechtering | 32 |

| **Multivariate Tests**^a^ | | | | | |
| --- | --- | --- | --- | --- | --- |
| Effect | | Value | F | Hypothesis df | Error df |
| factor1 | Pillai's Trace | ,179 | 15,876^b^ | 1,000 | 73,000 |
|  | Wilks' Lambda | ,821 | 15,876^b^ | 1,000 | 73,000 |
|  | Hotelling's Trace | ,217 | 15,876^b^ | 1,000 | 73,000 |
|  | Roy's Largest Root | ,217 | 15,876^b^ | 1,000 | 73,000 |
| factor1 * Barthel_beterofstabiel_slechter | Pillai's Trace | ,248 | 24,138^b^ | 1,000 | 73,000 |
|  | Wilks' Lambda | ,752 | 24,138^b^ | 1,000 | 73,000 |
|  | Hotelling's Trace | ,331 | 24,138^b^ | 1,000 | 73,000 |
|  | Roy's Largest Root | ,331 | 24,138^b^ | 1,000 | 73,000 |

| **Multivariate Tests**^a^ | | |  |  |  |
| --- | --- | --- | --- | --- | --- |
| Effect | | Sig. |  |  |  |
| factor1 | Pillai's Trace | <,001 |  |  |  |
|  | Wilks' Lambda | <,001 |  |  |  |
|  | Hotelling's Trace | <,001 |  |  |  |
|  | Roy's Largest Root | <,001 |  |  |  |
| factor1 * Barthel_beterofstabiel_slechter | Pillai's Trace | <,001 |  |  |  |
|  | Wilks' Lambda | <,001 |  |  |  |
|  | Hotelling's Trace | <,001 |  |  |  |
|  | Roy's Largest Root | <,001 |  |  |  |
|  |  |  |  |  |  |
|  |  |  |  |  |  |

| a. Design: Intercept + Barthel_beterofstabiel_slechter Within Subjects Design: factor1 |
| --- |
| b. Exact statistic |

| **Mauchly's Test of Sphericity**^a^ | | | | | |
| --- | --- | --- | --- | --- | --- |
| Measure: MEASURE_1 | | | | | |
| Within Subjects Effect | Mauchly's W | Approx. Chi-Square | df | Sig. | Epsilon^b^ |
|  |  |  |  |  | Greenhouse-Geisser |
| factor1 | 1,000 | ,000 | 0 | . | 1,000 |

| **Mauchly's Test of Sphericity**^a^ | | | |  |  |  |  |
| --- | --- | --- | --- | --- | --- | --- | --- |
| Measure: MEASURE_1 | | | |  |  |  |  |
| Within Subjects Effect | Epsilon | | |  |  |  |  |
|  | Huynh-Feldt | Lower-bound | |  |  |  |  |
| factor1 | 1,000 | 1,000 | |  |  |  |  |
|  |  | |  | |  |  |  |
|  |  | |  | |  |  |  |
|  |  | |  | |  |  |  |

| Tests the null hypothesis that the error covariance matrix of the orthonormalized transformed dependent variables is proportional to an identity matrix.^a^ |
| --- |
| a. Design: Intercept + Barthel_beterofstabiel_slechter Within Subjects Design: factor1 |
| b. May be used to adjust the degrees of freedom for the averaged tests of significance. Corrected tests are displayed in the Tests of Within-Subjects Effects table. |

| **Tests of Within-Subjects Effects** | | | | |
| --- | --- | --- | --- | --- |
| Measure: MEASURE_1 | | | | |
| Source | | Type III Sum of Squares | df | Mean Square |
| factor1 | Sphericity Assumed | 42,220 | 1 | 42,220 |
|  | Greenhouse-Geisser | 42,220 | 1,000 | 42,220 |
|  | Huynh-Feldt | 42,220 | 1,000 | 42,220 |
|  | Lower-bound | 42,220 | 1,000 | 42,220 |
| factor1 * Barthel_beterofstabiel_slechter | Sphericity Assumed | 64,194 | 1 | 64,194 |
|  | Greenhouse-Geisser | 64,194 | 1,000 | 64,194 |
|  | Huynh-Feldt | 64,194 | 1,000 | 64,194 |
|  | Lower-bound | 64,194 | 1,000 | 64,194 |
| Error(factor1) | Sphericity Assumed | 194,140 | 73 | 2,659 |
|  | Greenhouse-Geisser | 194,140 | 73,000 | 2,659 |
|  | Huynh-Feldt | 194,140 | 73,000 | 2,659 |
|  | Lower-bound | 194,140 | 73,000 | 2,659 |

| **Tests of Within-Subjects Effects** | | | |
| --- | --- | --- | --- |
| Measure: MEASURE_1 | | | |
| Source | | F | Sig. |
| factor1 | Sphericity Assumed | 15,876 | <,001 |
|  | Greenhouse-Geisser | 15,876 | <,001 |
|  | Huynh-Feldt | 15,876 | <,001 |
|  | Lower-bound | 15,876 | <,001 |
| factor1 * Barthel_beterofstabiel_slechter | Sphericity Assumed | 24,138 | <,001 |
|  | Greenhouse-Geisser | 24,138 | <,001 |
|  | Huynh-Feldt | 24,138 | <,001 |
|  | Lower-bound | 24,138 | <,001 |
| Error(factor1) | Sphericity Assumed |  |  |
|  | Greenhouse-Geisser |  |  |
|  | Huynh-Feldt |  |  |
|  | Lower-bound |  |  |

| **Tests of Within-Subjects Contrasts** | | | | | |
| --- | --- | --- | --- | --- | --- |
| Measure: MEASURE_1 | | | | | |
| Source | factor1 | Type III Sum of Squares | df | Mean Square | F |
| factor1 | Linear | 42,220 | 1 | 42,220 | 15,876 |
| factor1 * Barthel_beterofstabiel_slechter | Linear | 64,194 | 1 | 64,194 | 24,138 |
| Error(factor1) | Linear | 194,140 | 73 | 2,659 |  |

| **Tests of Within-Subjects Contrasts** | | |
| --- | --- | --- |
| Measure: MEASURE_1 | | |
| Source | factor1 | Sig. |
| factor1 | Linear | <,001 |
| factor1 * Barthel_beterofstabiel_slechter | Linear | <,001 |
| Error(factor1) | Linear |  |

| **Tests of Between-Subjects Effects** | | | | | |
| --- | --- | --- | --- | --- | --- |
| Measure: MEASURE_1 | | | | | |
| Transformed Variable: Average | | | | | |
| Source | Type III Sum of Squares | df | Mean Square | F | Sig. |
| Intercept | 3074,303 | 1 | 3074,303 | 132,636 | <,001 |
| Barthel_beterofstabiel_slechter | 12,809 | 1 | 12,809 | ,553 | ,460 |
| Error | 1692,031 | 73 | 23,179 |  |  |

**Profile Plots**

**General Linear Model**

| **Within-Subjects Factors** | |
| --- | --- |
| Measure: MEASURE_1 | |
| factor1 | Dependent Variable |
| 1 | m30s_CST_t0 |
| 2 | m30s_CST_tdbd |

| **Between-Subjects Factors** | | | |
| --- | --- | --- | --- |
|  | | Value Label | N |
| Barthel beter of stabiel-slechter | 1 | verbetering | 43 |
|  | 2 | stabiel of verslechtering | 32 |

| **Multivariate Tests**^a^ | | | | | |
| --- | --- | --- | --- | --- | --- |
| Effect | | Value | F | Hypothesis df | Error df |
| factor1 | Pillai's Trace | ,179 | 15,876^b^ | 1,000 | 73,000 |
|  | Wilks' Lambda | ,821 | 15,876^b^ | 1,000 | 73,000 |
|  | Hotelling's Trace | ,217 | 15,876^b^ | 1,000 | 73,000 |
|  | Roy's Largest Root | ,217 | 15,876^b^ | 1,000 | 73,000 |
| factor1 * Barthel_beterofstabiel_slechter | Pillai's Trace | ,248 | 24,138^b^ | 1,000 | 73,000 |
|  | Wilks' Lambda | ,752 | 24,138^b^ | 1,000 | 73,000 |
|  | Hotelling's Trace | ,331 | 24,138^b^ | 1,000 | 73,000 |
|  | Roy's Largest Root | ,331 | 24,138^b^ | 1,000 | 73,000 |

| **Multivariate Tests**^a^ | | |  |  |  |
| --- | --- | --- | --- | --- | --- |
| Effect | | Sig. |  |  |  |
| factor1 | Pillai's Trace | <,001 |  |  |  |
|  | Wilks' Lambda | <,001 |  |  |  |
|  | Hotelling's Trace | <,001 |  |  |  |
|  | Roy's Largest Root | <,001 |  |  |  |
| factor1 * Barthel_beterofstabiel_slechter | Pillai's Trace | <,001 |  |  |  |
|  | Wilks' Lambda | <,001 |  |  |  |
|  | Hotelling's Trace | <,001 |  |  |  |
|  | Roy's Largest Root | <,001 |  |  |  |
|  |  |  |  |  |  |
|  |  |  |  |  |  |

| a. Design: Intercept + Barthel_beterofstabiel_slechter Within Subjects Design: factor1 |
| --- |
| b. Exact statistic |

| **Mauchly's Test of Sphericity**^a^ | | | | | |
| --- | --- | --- | --- | --- | --- |
| Measure: MEASURE_1 | | | | | |
| Within Subjects Effect | Mauchly's W | Approx. Chi-Square | df | Sig. | Epsilon^b^ |
|  |  |  |  |  | Greenhouse-Geisser |
| factor1 | 1,000 | ,000 | 0 | . | 1,000 |

| **Mauchly's Test of Sphericity**^a^ | | | |  |  |  |  |
| --- | --- | --- | --- | --- | --- | --- | --- |
| Measure: MEASURE_1 | | | |  |  |  |  |
| Within Subjects Effect | Epsilon | | |  |  |  |  |
|  | Huynh-Feldt | Lower-bound | |  |  |  |  |
| factor1 | 1,000 | 1,000 | |  |  |  |  |
|  |  | |  | |  |  |  |
|  |  | |  | |  |  |  |
|  |  | |  | |  |  |  |

| Tests the null hypothesis that the error covariance matrix of the orthonormalized transformed dependent variables is proportional to an identity matrix.^a^ |
| --- |
| a. Design: Intercept + Barthel_beterofstabiel_slechter Within Subjects Design: factor1 |
| b. May be used to adjust the degrees of freedom for the averaged tests of significance. Corrected tests are displayed in the Tests of Within-Subjects Effects table. |

| **Tests of Within-Subjects Effects** | | | | |
| --- | --- | --- | --- | --- |
| Measure: MEASURE_1 | | | | |
| Source | | Type III Sum of Squares | df | Mean Square |
| factor1 | Sphericity Assumed | 42,220 | 1 | 42,220 |
|  | Greenhouse-Geisser | 42,220 | 1,000 | 42,220 |
|  | Huynh-Feldt | 42,220 | 1,000 | 42,220 |
|  | Lower-bound | 42,220 | 1,000 | 42,220 |
| factor1 * Barthel_beterofstabiel_slechter | Sphericity Assumed | 64,194 | 1 | 64,194 |
|  | Greenhouse-Geisser | 64,194 | 1,000 | 64,194 |
|  | Huynh-Feldt | 64,194 | 1,000 | 64,194 |
|  | Lower-bound | 64,194 | 1,000 | 64,194 |
| Error(factor1) | Sphericity Assumed | 194,140 | 73 | 2,659 |
|  | Greenhouse-Geisser | 194,140 | 73,000 | 2,659 |
|  | Huynh-Feldt | 194,140 | 73,000 | 2,659 |
|  | Lower-bound | 194,140 | 73,000 | 2,659 |

| **Tests of Within-Subjects Effects** | | | |
| --- | --- | --- | --- |
| Measure: MEASURE_1 | | | |
| Source | | F | Sig. |
| factor1 | Sphericity Assumed | 15,876 | <,001 |
|  | Greenhouse-Geisser | 15,876 | <,001 |
|  | Huynh-Feldt | 15,876 | <,001 |
|  | Lower-bound | 15,876 | <,001 |
| factor1 * Barthel_beterofstabiel_slechter | Sphericity Assumed | 24,138 | <,001 |
|  | Greenhouse-Geisser | 24,138 | <,001 |
|  | Huynh-Feldt | 24,138 | <,001 |
|  | Lower-bound | 24,138 | <,001 |
| Error(factor1) | Sphericity Assumed |  |  |
|  | Greenhouse-Geisser |  |  |
|  | Huynh-Feldt |  |  |
|  | Lower-bound |  |  |

| **Tests of Within-Subjects Contrasts** | | | | | |
| --- | --- | --- | --- | --- | --- |
| Measure: MEASURE_1 | | | | | |
| Source | factor1 | Type III Sum of Squares | df | Mean Square | F |
| factor1 | Linear | 42,220 | 1 | 42,220 | 15,876 |
| factor1 * Barthel_beterofstabiel_slechter | Linear | 64,194 | 1 | 64,194 | 24,138 |
| Error(factor1) | Linear | 194,140 | 73 | 2,659 |  |

| **Tests of Within-Subjects Contrasts** | | |
| --- | --- | --- |
| Measure: MEASURE_1 | | |
| Source | factor1 | Sig. |
| factor1 | Linear | <,001 |
| factor1 * Barthel_beterofstabiel_slechter | Linear | <,001 |
| Error(factor1) | Linear |  |

| **Tests of Between-Subjects Effects** | | | | | |
| --- | --- | --- | --- | --- | --- |
| Measure: MEASURE_1 | | | | | |
| Transformed Variable: Average | | | | | |
| Source | Type III Sum of Squares | df | Mean Square | F | Sig. |
| Intercept | 3074,303 | 1 | 3074,303 | 132,636 | <,001 |
| Barthel_beterofstabiel_slechter | 12,809 | 1 | 12,809 | ,553 | ,460 |
| Error | 1692,031 | 73 | 23,179 |  |  |

**Profile Plots**

**General Linear Model**

| **Within-Subjects Factors** | |
| --- | --- |
| Measure: MEASURE_1 | |
| factor1 | Dependent Variable |
| 1 | HGS_Jamar_t0 |
| 2 | HGS_Jamar_tdbd |

| **Between-Subjects Factors** | | | |
| --- | --- | --- | --- |
|  | | Value Label | N |
| Barthel beter of stabiel-slechter | 1 | verbetering | 43 |
|  | 2 | stabiel of verslechtering | 31 |

| **Multivariate Tests**^a^ | | | | | |
| --- | --- | --- | --- | --- | --- |
| Effect | | Value | F | Hypothesis df | Error df |
| factor1 | Pillai's Trace | ,020 | 1,449^b^ | 1,000 | 72,000 |
|  | Wilks' Lambda | ,980 | 1,449^b^ | 1,000 | 72,000 |
|  | Hotelling's Trace | ,020 | 1,449^b^ | 1,000 | 72,000 |
|  | Roy's Largest Root | ,020 | 1,449^b^ | 1,000 | 72,000 |
| factor1 * Barthel_beterofstabiel_slechter | Pillai's Trace | ,045 | 3,427^b^ | 1,000 | 72,000 |
|  | Wilks' Lambda | ,955 | 3,427^b^ | 1,000 | 72,000 |
|  | Hotelling's Trace | ,048 | 3,427^b^ | 1,000 | 72,000 |
|  | Roy's Largest Root | ,048 | 3,427^b^ | 1,000 | 72,000 |

| **Multivariate Tests**^a^ | | |  |  |  |
| --- | --- | --- | --- | --- | --- |
| Effect | | Sig. |  |  |  |
| factor1 | Pillai's Trace | ,233 |  |  |  |
|  | Wilks' Lambda | ,233 |  |  |  |
|  | Hotelling's Trace | ,233 |  |  |  |
|  | Roy's Largest Root | ,233 |  |  |  |
| factor1 * Barthel_beterofstabiel_slechter | Pillai's Trace | ,068 |  |  |  |
|  | Wilks' Lambda | ,068 |  |  |  |
|  | Hotelling's Trace | ,068 |  |  |  |
|  | Roy's Largest Root | ,068 |  |  |  |
|  |  |  |  |  |  |
|  |  |  |  |  |  |

| a. Design: Intercept + Barthel_beterofstabiel_slechter Within Subjects Design: factor1 |
| --- |
| b. Exact statistic |

| **Mauchly's Test of Sphericity**^a^ | | | | | |
| --- | --- | --- | --- | --- | --- |
| Measure: MEASURE_1 | | | | | |
| Within Subjects Effect | Mauchly's W | Approx. Chi-Square | df | Sig. | Epsilon^b^ |
|  |  |  |  |  | Greenhouse-Geisser |
| factor1 | 1,000 | ,000 | 0 | . | 1,000 |

| **Mauchly's Test of Sphericity**^a^ | | | |  |  |  |  |
| --- | --- | --- | --- | --- | --- | --- | --- |
| Measure: MEASURE_1 | | | |  |  |  |  |
| Within Subjects Effect | Epsilon | | |  |  |  |  |
|  | Huynh-Feldt | Lower-bound | |  |  |  |  |
| factor1 | 1,000 | 1,000 | |  |  |  |  |
|  |  | |  | |  |  |  |
|  |  | |  | |  |  |  |
|  |  | |  | |  |  |  |

| Tests the null hypothesis that the error covariance matrix of the orthonormalized transformed dependent variables is proportional to an identity matrix.^a^ |
| --- |
| a. Design: Intercept + Barthel_beterofstabiel_slechter Within Subjects Design: factor1 |
| b. May be used to adjust the degrees of freedom for the averaged tests of significance. Corrected tests are displayed in the Tests of Within-Subjects Effects table. |

| **Tests of Within-Subjects Effects** | | | | |
| --- | --- | --- | --- | --- |
| Measure: MEASURE_1 | | | | |
| Source | | Type III Sum of Squares | df | Mean Square |
| factor1 | Sphericity Assumed | 15,683 | 1 | 15,683 |
|  | Greenhouse-Geisser | 15,683 | 1,000 | 15,683 |
|  | Huynh-Feldt | 15,683 | 1,000 | 15,683 |
|  | Lower-bound | 15,683 | 1,000 | 15,683 |
| factor1 * Barthel_beterofstabiel_slechter | Sphericity Assumed | 37,089 | 1 | 37,089 |
|  | Greenhouse-Geisser | 37,089 | 1,000 | 37,089 |
|  | Huynh-Feldt | 37,089 | 1,000 | 37,089 |
|  | Lower-bound | 37,089 | 1,000 | 37,089 |
| Error(factor1) | Sphericity Assumed | 779,269 | 72 | 10,823 |
|  | Greenhouse-Geisser | 779,269 | 72,000 | 10,823 |
|  | Huynh-Feldt | 779,269 | 72,000 | 10,823 |
|  | Lower-bound | 779,269 | 72,000 | 10,823 |

| **Tests of Within-Subjects Effects** | | | |
| --- | --- | --- | --- |
| Measure: MEASURE_1 | | | |
| Source | | F | Sig. |
| factor1 | Sphericity Assumed | 1,449 | ,233 |
|  | Greenhouse-Geisser | 1,449 | ,233 |
|  | Huynh-Feldt | 1,449 | ,233 |
|  | Lower-bound | 1,449 | ,233 |
| factor1 * Barthel_beterofstabiel_slechter | Sphericity Assumed | 3,427 | ,068 |
|  | Greenhouse-Geisser | 3,427 | ,068 |
|  | Huynh-Feldt | 3,427 | ,068 |
|  | Lower-bound | 3,427 | ,068 |
| Error(factor1) | Sphericity Assumed |  |  |
|  | Greenhouse-Geisser |  |  |
|  | Huynh-Feldt |  |  |
|  | Lower-bound |  |  |

| **Tests of Within-Subjects Contrasts** | | | | | |
| --- | --- | --- | --- | --- | --- |
| Measure: MEASURE_1 | | | | | |
| Source | factor1 | Type III Sum of Squares | df | Mean Square | F |
| factor1 | Linear | 15,683 | 1 | 15,683 | 1,449 |
| factor1 * Barthel_beterofstabiel_slechter | Linear | 37,089 | 1 | 37,089 | 3,427 |
| Error(factor1) | Linear | 779,269 | 72 | 10,823 |  |

| **Tests of Within-Subjects Contrasts** | | |
| --- | --- | --- |
| Measure: MEASURE_1 | | |
| Source | factor1 | Sig. |
| factor1 | Linear | ,233 |
| factor1 * Barthel_beterofstabiel_slechter | Linear | ,068 |
| Error(factor1) | Linear |  |

| **Tests of Between-Subjects Effects** | | | | | |
| --- | --- | --- | --- | --- | --- |
| Measure: MEASURE_1 | | | | | |
| Transformed Variable: Average | | | | | |
| Source | Type III Sum of Squares | df | Mean Square | F | Sig. |
| Intercept | 45924,577 | 1 | 45924,577 | 247,620 | <,001 |
| Barthel_beterofstabiel_slechter | 12,415 | 1 | 12,415 | ,067 | ,797 |
| Error | 13353,403 | 72 | 185,464 |  |  |

**Profile Plots**

**General Linear Model**

| **Within-Subjects Factors** | |
| --- | --- |
| Measure: MEASURE_1 | |
| factor1 | Dependent Variable |
| 1 | HGS_Jamar_t0 |
| 2 | HGS_Jamar_tdbd |

| **Between-Subjects Factors** | | | |
| --- | --- | --- | --- |
|  | | Value Label | N |
| SPPB2 verandering (1=beter 2= stabiel of slechter) | 1 | beter | 41 |
|  | 2 | stabiel of slechter | 34 |

| **Multivariate Tests**^a^ | | | | | |
| --- | --- | --- | --- | --- | --- |
| Effect | | Value | F | Hypothesis df | Error df |
| factor1 | Pillai's Trace | ,028 | 2,134^b^ | 1,000 | 73,000 |
|  | Wilks' Lambda | ,972 | 2,134^b^ | 1,000 | 73,000 |
|  | Hotelling's Trace | ,029 | 2,134^b^ | 1,000 | 73,000 |
|  | Roy's Largest Root | ,029 | 2,134^b^ | 1,000 | 73,000 |
| factor1 * SPPB_change2 | Pillai's Trace | ,044 | 3,396^b^ | 1,000 | 73,000 |
|  | Wilks' Lambda | ,956 | 3,396^b^ | 1,000 | 73,000 |
|  | Hotelling's Trace | ,047 | 3,396^b^ | 1,000 | 73,000 |
|  | Roy's Largest Root | ,047 | 3,396^b^ | 1,000 | 73,000 |

| **Multivariate Tests**^a^ | | |  |  |  |
| --- | --- | --- | --- | --- | --- |
| Effect | | Sig. |  |  |  |
| factor1 | Pillai's Trace | ,148 |  |  |  |
|  | Wilks' Lambda | ,148 |  |  |  |
|  | Hotelling's Trace | ,148 |  |  |  |
|  | Roy's Largest Root | ,148 |  |  |  |
| factor1 * SPPB_change2 | Pillai's Trace | ,069 |  |  |  |
|  | Wilks' Lambda | ,069 |  |  |  |
|  | Hotelling's Trace | ,069 |  |  |  |
|  | Roy's Largest Root | ,069 |  |  |  |
|  |  |  |  |  |  |
|  |  |  |  |  |  |

| a. Design: Intercept + SPPB_change2 Within Subjects Design: factor1 |
| --- |
| b. Exact statistic |

| **Mauchly's Test of Sphericity**^a^ | | | | | |
| --- | --- | --- | --- | --- | --- |
| Measure: MEASURE_1 | | | | | |
| Within Subjects Effect | Mauchly's W | Approx. Chi-Square | df | Sig. | Epsilon^b^ |
|  |  |  |  |  | Greenhouse-Geisser |
| factor1 | 1,000 | ,000 | 0 | . | 1,000 |

| **Mauchly's Test of Sphericity**^a^ | | | |  |  |  |  |
| --- | --- | --- | --- | --- | --- | --- | --- |
| Measure: MEASURE_1 | | | |  |  |  |  |
| Within Subjects Effect | Epsilon | | |  |  |  |  |
|  | Huynh-Feldt | Lower-bound | |  |  |  |  |
| factor1 | 1,000 | 1,000 | |  |  |  |  |
|  |  | |  | |  |  |  |
|  |  | |  | |  |  |  |
|  |  | |  | |  |  |  |

| Tests the null hypothesis that the error covariance matrix of the orthonormalized transformed dependent variables is proportional to an identity matrix.^a^ |
| --- |
| a. Design: Intercept + SPPB_change2 Within Subjects Design: factor1 |
| b. May be used to adjust the degrees of freedom for the averaged tests of significance. Corrected tests are displayed in the Tests of Within-Subjects Effects table. |

| **Tests of Within-Subjects Effects** | | | | |
| --- | --- | --- | --- | --- |
| Measure: MEASURE_1 | | | | |
| Source | | Type III Sum of Squares | df | Mean Square |
| factor1 | Sphericity Assumed | 23,042 | 1 | 23,042 |
|  | Greenhouse-Geisser | 23,042 | 1,000 | 23,042 |
|  | Huynh-Feldt | 23,042 | 1,000 | 23,042 |
|  | Lower-bound | 23,042 | 1,000 | 23,042 |
| factor1 * SPPB_change2 | Sphericity Assumed | 36,668 | 1 | 36,668 |
|  | Greenhouse-Geisser | 36,668 | 1,000 | 36,668 |
|  | Huynh-Feldt | 36,668 | 1,000 | 36,668 |
|  | Lower-bound | 36,668 | 1,000 | 36,668 |
| Error(factor1) | Sphericity Assumed | 788,292 | 73 | 10,799 |
|  | Greenhouse-Geisser | 788,292 | 73,000 | 10,799 |
|  | Huynh-Feldt | 788,292 | 73,000 | 10,799 |
|  | Lower-bound | 788,292 | 73,000 | 10,799 |

| **Tests of Within-Subjects Effects** | | | |
| --- | --- | --- | --- |
| Measure: MEASURE_1 | | | |
| Source | | F | Sig. |
| factor1 | Sphericity Assumed | 2,134 | ,148 |
|  | Greenhouse-Geisser | 2,134 | ,148 |
|  | Huynh-Feldt | 2,134 | ,148 |
|  | Lower-bound | 2,134 | ,148 |
| factor1 * SPPB_change2 | Sphericity Assumed | 3,396 | ,069 |
|  | Greenhouse-Geisser | 3,396 | ,069 |
|  | Huynh-Feldt | 3,396 | ,069 |
|  | Lower-bound | 3,396 | ,069 |
| Error(factor1) | Sphericity Assumed |  |  |
|  | Greenhouse-Geisser |  |  |
|  | Huynh-Feldt |  |  |
|  | Lower-bound |  |  |

| **Tests of Within-Subjects Contrasts** | | | | | | |
| --- | --- | --- | --- | --- | --- | --- |
| Measure: MEASURE_1 | | | | | | |
| Source | factor1 | Type III Sum of Squares | df | Mean Square | F | Sig. |
| factor1 | Linear | 23,042 | 1 | 23,042 | 2,134 | ,148 |
| factor1 * SPPB_change2 | Linear | 36,668 | 1 | 36,668 | 3,396 | ,069 |
| Error(factor1) | Linear | 788,292 | 73 | 10,799 |  |  |

| **Tests of Between-Subjects Effects** | | | | | |
| --- | --- | --- | --- | --- | --- |
| Measure: MEASURE_1 | | | | | |
| Transformed Variable: Average | | | | | |
| Source | Type III Sum of Squares | df | Mean Square | F | Sig. |
| Intercept | 45269,861 | 1 | 45269,861 | 253,452 | <,001 |
| SPPB_change2 | 625,781 | 1 | 625,781 | 3,504 | ,065 |
| Error | 13038,779 | 73 | 178,613 |  |  |

**Profile Plots**

**General Linear Model**

| **Within-Subjects Factors** | |
| --- | --- |
| Measure: MEASURE_1 | |
| factor1 | Dependent Variable |
| 1 | m30s_CST_t0 |
| 2 | m30s_CST_tdbd |

| **Between-Subjects Factors** | | | |
| --- | --- | --- | --- |
|  | | Value Label | N |
| SPPB2 verandering (1=beter 2= stabiel of slechter) | 1 | beter | 41 |
|  | 2 | stabiel of slechter | 34 |

| **Multivariate Tests**^a^ | | | | | |
| --- | --- | --- | --- | --- | --- |
| Effect | | Value | F | Hypothesis df | Error df |
| factor1 | Pillai's Trace | ,189 | 17,055^b^ | 1,000 | 73,000 |
|  | Wilks' Lambda | ,811 | 17,055^b^ | 1,000 | 73,000 |
|  | Hotelling's Trace | ,234 | 17,055^b^ | 1,000 | 73,000 |
|  | Roy's Largest Root | ,234 | 17,055^b^ | 1,000 | 73,000 |
| factor1 * SPPB_change2 | Pillai's Trace | ,159 | 13,794^b^ | 1,000 | 73,000 |
|  | Wilks' Lambda | ,841 | 13,794^b^ | 1,000 | 73,000 |
|  | Hotelling's Trace | ,189 | 13,794^b^ | 1,000 | 73,000 |
|  | Roy's Largest Root | ,189 | 13,794^b^ | 1,000 | 73,000 |

| **Multivariate Tests**^a^ | | |  |  |  |
| --- | --- | --- | --- | --- | --- |
| Effect | | Sig. |  |  |  |
| factor1 | Pillai's Trace | <,001 |  |  |  |
|  | Wilks' Lambda | <,001 |  |  |  |
|  | Hotelling's Trace | <,001 |  |  |  |
|  | Roy's Largest Root | <,001 |  |  |  |
| factor1 * SPPB_change2 | Pillai's Trace | <,001 |  |  |  |
|  | Wilks' Lambda | <,001 |  |  |  |
|  | Hotelling's Trace | <,001 |  |  |  |
|  | Roy's Largest Root | <,001 |  |  |  |
|  |  |  |  |  |  |
|  |  |  |  |  |  |

| a. Design: Intercept + SPPB_change2 Within Subjects Design: factor1 |
| --- |
| b. Exact statistic |

| **Mauchly's Test of Sphericity**^a^ | | | | | |
| --- | --- | --- | --- | --- | --- |
| Measure: MEASURE_1 | | | | | |
| Within Subjects Effect | Mauchly's W | Approx. Chi-Square | df | Sig. | Epsilon^b^ |
|  |  |  |  |  | Greenhouse-Geisser |
| factor1 | 1,000 | ,000 | 0 | . | 1,000 |

| **Mauchly's Test of Sphericity**^a^ | | | |  |  |  |  |
| --- | --- | --- | --- | --- | --- | --- | --- |
| Measure: MEASURE_1 | | | |  |  |  |  |
| Within Subjects Effect | Epsilon | | |  |  |  |  |
|  | Huynh-Feldt | Lower-bound | |  |  |  |  |
| factor1 | 1,000 | 1,000 | |  |  |  |  |
|  |  | |  | |  |  |  |
|  |  | |  | |  |  |  |
|  |  | |  | |  |  |  |

| Tests the null hypothesis that the error covariance matrix of the orthonormalized transformed dependent variables is proportional to an identity matrix.^a^ |
| --- |
| a. Design: Intercept + SPPB_change2 Within Subjects Design: factor1 |
| b. May be used to adjust the degrees of freedom for the averaged tests of significance. Corrected tests are displayed in the Tests of Within-Subjects Effects table. |

| **Tests of Within-Subjects Effects** | | | | |
| --- | --- | --- | --- | --- |
| Measure: MEASURE_1 | | | | |
| Source | | Type III Sum of Squares | df | Mean Square |
| factor1 | Sphericity Assumed | 50,763 | 1 | 50,763 |
|  | Greenhouse-Geisser | 50,763 | 1,000 | 50,763 |
|  | Huynh-Feldt | 50,763 | 1,000 | 50,763 |
|  | Lower-bound | 50,763 | 1,000 | 50,763 |
| factor1 * SPPB_change2 | Sphericity Assumed | 41,056 | 1 | 41,056 |
|  | Greenhouse-Geisser | 41,056 | 1,000 | 41,056 |
|  | Huynh-Feldt | 41,056 | 1,000 | 41,056 |
|  | Lower-bound | 41,056 | 1,000 | 41,056 |
| Error(factor1) | Sphericity Assumed | 217,277 | 73 | 2,976 |
|  | Greenhouse-Geisser | 217,277 | 73,000 | 2,976 |
|  | Huynh-Feldt | 217,277 | 73,000 | 2,976 |
|  | Lower-bound | 217,277 | 73,000 | 2,976 |

| **Tests of Within-Subjects Effects** | | | |
| --- | --- | --- | --- |
| Measure: MEASURE_1 | | | |
| Source | | F | Sig. |
| factor1 | Sphericity Assumed | 17,055 | <,001 |
|  | Greenhouse-Geisser | 17,055 | <,001 |
|  | Huynh-Feldt | 17,055 | <,001 |
|  | Lower-bound | 17,055 | <,001 |
| factor1 * SPPB_change2 | Sphericity Assumed | 13,794 | <,001 |
|  | Greenhouse-Geisser | 13,794 | <,001 |
|  | Huynh-Feldt | 13,794 | <,001 |
|  | Lower-bound | 13,794 | <,001 |
| Error(factor1) | Sphericity Assumed |  |  |
|  | Greenhouse-Geisser |  |  |
|  | Huynh-Feldt |  |  |
|  | Lower-bound |  |  |

| **Tests of Within-Subjects Contrasts** | | | | | | |
| --- | --- | --- | --- | --- | --- | --- |
| Measure: MEASURE_1 | | | | | | |
| Source | factor1 | Type III Sum of Squares | df | Mean Square | F | Sig. |
| factor1 | Linear | 50,763 | 1 | 50,763 | 17,055 | <,001 |
| factor1 * SPPB_change2 | Linear | 41,056 | 1 | 41,056 | 13,794 | <,001 |
| Error(factor1) | Linear | 217,277 | 73 | 2,976 |  |  |

| **Tests of Between-Subjects Effects** | | | | | |
| --- | --- | --- | --- | --- | --- |
| Measure: MEASURE_1 | | | | | |
| Transformed Variable: Average | | | | | |
| Source | Type III Sum of Squares | df | Mean Square | F | Sig. |
| Intercept | 3139,093 | 1 | 3139,093 | 135,281 | <,001 |
| SPPB_change2 | 10,933 | 1 | 10,933 | ,471 | ,495 |
| Error | 1693,907 | 73 | 23,204 |  |  |

**Profile Plots**

Data written to C:\Users\151390\OneDrive - Zuyderland\walther\onderzoek\CST\prognose m-30s-CST\Plos One\Minimal data.xlsx.
90 variables and 92 cases written to range: SPSS.
Variable: PatientNr Type: Number Width: 2 Dec: 0
Variable: Gender Type: Number Width: 8 Dec: 0
Variable: SNAQ Type: Number Width: 1 Dec: 0
Variable: BMI Type: Number Width: 4 Dec: 1
Variable: GFI Type: Number Width: 2 Dec: 0
Variable: KATZ_ADL Type: Number Width: 1 Dec: 0
Variable: CCI Type: Number Width: 2 Dec: 0
Variable: Admission_diagnosis Type: String Width: 92
Variable: Age Type: Number Width: 2 Dec: 0
Variable: BI_bowel_t0 Type: Number Width: 1 Dec: 0
Variable: BI_bladder_t0 Type: Number Width: 1 Dec: 0
Variable: BI_selfcare_t0 Type: Number Width: 1 Dec: 0
Variable: BI_toilet_t0 Type: Number Width: 1 Dec: 0
Variable: BI_eating_t0 Type: Number Width: 1 Dec: 0
Variable: BI_transfers_t0 Type: Number Width: 1 Dec: 0
Variable: BI_mobility_t0 Type: Number Width: 1 Dec: 0
Variable: BI_changing_t0 Type: Number Width: 1 Dec: 0
Variable: BI_stair_t0 Type: Number Width: 1 Dec: 0
Variable: BI_shower_t0 Type: Number Width: 1 Dec: 0
Variable: Barthel_Index_total_t00 Type: Number Width: 2 Dec: 0
Variable: m30s_CST_t0 Type: Number Width: 2 Dec: 0
Variable: M30CST_cat2 Type: Number Width: 8 Dec: 2
Variable: M30CST_cat3 Type: Number Width: 8 Dec: 0
Variable: M30CST_cat4 Type: Number Width: 8 Dec: 0
Variable: M30CST_CAT6 Type: Number Width: 8 Dec: 0
Variable: M30CST_CAT5 Type: Number Width: 8 Dec: 0
Variable: SPPB_balance_t0 Type: Number Width: 1 Dec: 0
Variable: SPPB_gaitspeed_t0 Type: Number Width: 1 Dec: 0
Variable: SPPB_5t_chairstand_t0 Type: Number Width: 1 Dec: 0
Variable: CST_SPPB_cat2 Type: Number Width: 8 Dec: 0
Variable: CST_SPPB Type: Number Width: 8 Dec: 0
Variable: SPPB_total_t0 Type: Number Width: 1 Dec: 0
Variable: HGS_Jamar_t0 Type: Number Width: 2 Dec: 0
Variable: BMI_ Type: Number Width: 4 Dec: 1
Variable: HKK_EWGSOP_BMI Type: Number Width: 8 Dec: 0
Variable: HKK_EWGSOP_2 Type: Number Width: 8 Dec: 0
Variable: HGS_Vigori_t0 Type: Number Width: 2 Dec: 0
Variable: m30s_CST_td7 Type: Number Width: 2 Dec: 0
Variable: m30s_CST_tdbd Type: Number Width: 23 Dec: 0
Variable: BI_bowel_tdbd Type: Number Width: 1 Dec: 0
Variable: BI_bladder_tdbd Type: Number Width: 1 Dec: 0
Variable: BI_selfcare_tdbd Type: Number Width: 1 Dec: 0
Variable: BI_toilet_tdbd Type: Number Width: 1 Dec: 0
Variable: BI_eating_tdbd Type: Number Width: 1 Dec: 0
Variable: BI_transfers_tdbd Type: Number Width: 1 Dec: 0
Variable: BI_mobility_tdbd Type: Number Width: 1 Dec: 0
Variable: BI_changing_tdbd Type: Number Width: 1 Dec: 0
Variable: BI_stair_tdbd Type: Number Width: 1 Dec: 0
Variable: BI_shower_tdbd Type: Number Width: 1 Dec: 0
Variable: Barthel_Index_tdbd Type: Number Width: 2 Dec: 0
Variable: SPPB_balance_tdbd Type: Number Width: 1 Dec: 0
Variable: SPPB_gaitspeed_tdbd Type: Number Width: 1 Dec: 0
Variable: SPPB_5tCST_tdbd Type: Number Width: 1 Dec: 0
Variable: SPPB_tdbd Type: Number Width: 2 Dec: 0
Variable: m30s_CST_tdod Type: Number Width: 2 Dec: 0
Variable: HGS_Jamar_tdbd Type: Number Width: 33 Dec: 0
Variable: HGS_Vigori_tdbd Type: Number Width: 2 Dec: 0
Variable: Opnameduur Type: Number Width: 8 Dec: 0
Variable: Sterfte_1mnd Type: Number Width: 8 Dec: 0
Variable: Sterfte_3mnd Type: Number Width: 8 Dec: 0
Variable: Sterfte_6mndnn Type: Number Width: 8 Dec: 0
Variable: Sterfte_6mnd Type: Number Width: 8 Dec: 0
Variable: PROG_DEATH_DEADD_NUM_1 Type: Number Width: 3 Dec: 0
Variable: dagentoteindestudieofoverlijden Type: Number Width: 8 Dec: 0
Variable: tijd3mnddoodoflevend Type: Number Width: 8 Dec: 0
Variable: Change_Barthel_Index_score Type: Number Width: 8 Dec: 2
Variable: Barthel_Index_verandering Type: Number Width: 8 Dec: 0
Variable: Barthel123 Type: Number Width: 8 Dec: 0
Variable: Barthel_beterofstabiel_slechter Type: Number Width: 8 Dec: 0
Variable: SUR_1 Type: Number Width: 10 Dec: 5
Variable: HAZ_1 Type: Number Width: 10 Dec: 5
Variable: SE_1 Type: Number Width: 10 Dec: 5
Variable: CUM_1 Type: Number Width: 10 Dec: 5
Variable: SUR_2 Type: Number Width: 10 Dec: 5
Variable: HAZ_2 Type: Number Width: 10 Dec: 5
Variable: SE_2 Type: Number Width: 10 Dec: 5
Variable: CUM_2 Type: Number Width: 10 Dec: 5
Variable: filter_$ Type: Number Width: 1 Dec: 0
Variable: Change_SPPB Type: Number Width: 8 Dec: 0
Variable: SPPB_change2 Type: Number Width: 8 Dec: 0
Variable: SPPB_change1 Type: Number Width: 8 Dec: 0
Variable: Change_m30sCST Type: Number Width: 8 Dec: 0
Variable: Change_m30sCSTcat Type: Number Width: 8 Dec: 0
Variable: dagentotoverlijden Type: Number Width: 8 Dec: 0
Variable: dagentotoverlijden2jaar Type: Number Width: 8 Dec: 0
Variable: overlijden_binnen_halfjaar Type: Number Width: 8 Dec: 0
Variable: overlijden_binnen_eenjaar Type: Number Width: 8 Dec: 0
Variable: overlijden_binnen_tweejaar Type: Number Width: 8 Dec: 0
Variable: Change_HGS Type: Number Width: 8 Dec: 0
Variable: Ghange_HGS_cat Type: Number Width: 8 Dec: 0
