# Supplementary material for: The modified 30-second chair stand test (m-30s-CST) is more sensitive than handgrip strength in detecting muscle strength changes and predicting physical performance in hospitalized geriatric patients
Source: PLoS One. 2026 Mar 16;21(3):e0331155. doi: 10.1371/journal.pone.0331155 (PMC12991214; doi:10.1371/journal.pone.0331155)
Supplement: Attachment — Submitted filename: Response to Reviewers final dd 24012026.docx [file pone.0331155.s007.docx]

## Ref. No.: PONE-D-2542511

Title: The modified 30-second chair stand test (m-30s-CST) is more sensitive than handgrip strength in detecting muscle strength changes and predicting physical performance in hospitalized geriatric patients

Author(s): Walther M.W.H. Sipers*; Martijn J.A. Rothbauer; Isis Ensink; Shannon Röhlinger; Audrey H.H. Merry

Corresponding Author: Walther M.W.H. Sipers

* First and last author

Affiliation of Corresponding Author: Zuyderland Medical Center

January 24^th^, 2025

Dear Editor,

We thank you/the academic editor and reviewers for evaluating the manuscript and for the feedback we have received for our manuscript. We thank you for giving us the opportunity for a revision. We have revised the manuscript according to the suggestions given and address the comments in a point-to-point fashion below. All changes in the manuscript and responses in this letter have been highlighted in red font.

**Academic Editor**

*The study addresses an important and clinically relevant question in geriatric hospital care and is clearly written. However, several methodological and analytical issues need to be addressed before the manuscript can be considered for publication. In particular, the limited sample size, potential selection bias, lack of adjustment for key confounders, and the empirical definition of m-30s-CST cut-offs weaken the strength of the conclusions. A major revision is required to improve the statistical robustness, clarify the definition of functional outcomes, and better highlight the clinical implications for geriatric practice.*

Thank you for sharing your honest and clear opinion. We agree that the CST parameter is for measuring strength according the EWGSOP2

**First comment:** Limited sample size

*We acknowledge this limitation, as also mentioned by reviewer #2. Please read our response at reviewer #2, first comment.*

**Second comment:** Potential selection bias

*We acknowledge this limitation, as also mentioned by reviewer #2. Please read our response at reviewer #2, second comment.*

**Third comment:** Lack of adjustment of key confounders

*We acknowledge this limitation, as also mentioned by reviewer #2. Please read our response at reviewer #2, forth comment.*

**Fourth comment:** m-30s-CST cut-off / definition of functional outcomes

*We acknowledge this limitation, as also mentioned by reviewer #2. Please read our response at reviewer #2, third comment.*

**Fifth comment:** highlighting the clinical implications

*We acknowledge this point of comment, as also mentioned by reviewer #2. Please read our response at reviewer #2, eighth comment.*

We thank you for clarifying that the point we are trying to make in the second paragraph of the discussion is not clearly connected to the rest of the content.

We addressed this issue in the discussion section by rewriting the discussion section and by adding a conclusion section with a clinical key message

**Comments of the reviewers**

**Reviewer #1:**

The manuscript is interesting but I have some concern about the clinical status of the patients.

First Comment: *Frailty: you are considered only physical frailty and not multidimensional frailty. Please see and discuss: Liguori I et al. Validation of "(fr)AGILE": a quick tool to identify multidimensional frailty in the elderly. BMC Geriatr. 2020 Sep 29;20(1):375.*

We thank the reviewer for the comment. We agree with the reviewer that in geriatric patients, it is important to consider more than just physical frailty. The reviewer suggests considering multi-domain frailty and refers to the article by Ligouri et al.: *Validation of (fr)agile: a quick tool to identify multidimensional frailty in the elderly* *(BMC Geriatrics 2020 Sep 29;20(1):375)*. In our study, we used the Groningen Frailty Indicator (GFI) that cover various domains, including physical, functional, psychological and social aspects. The GFI ranges from 0 to 15; a score of 4 or higher indicates frailty (*Bielderman A, van der Schans CP, van Lieshout MR, de Greef MH, Boersma F, Krijnen WP, et al. Multidimensional structure of the Groningen Frailty Indicator in community-dwelling older people. BMC Geriatr, 2013. 13:86 and Peters LL, Boter H, Buskens E, Slaets JP. Measurement properties of the Groningen Frailty Indicator in home-dwelling and institutionalized elderly people. Journal of the American Medical Directors Association 2012;13(6):546-51*). In our study, all patients were frail according to the GFI (mean GFI score of 6.1± 2.9 SD, line 200-201), but we were primarily interested in the relationship between changes in muscle strength measured by handgrip strength and leg strength using the m-30s-CST and physical functioning expressed as the SPPB and ADL-Barthel Index. It is possible that a change in cognitive functioning, for example, due to recovery from delirium, could also impact the aforementioned relationships. However, we did not specifically examine this in our study. This should be considered as a potential confounder and therefore it has been included in the discussion section and should be addressed in a future study.

Changes/additions in the manuscript:

1) Section ‘Materials and methods’ subsection ‘*Patients’ characteristics’*

*The frailty score was assessed according to the Groningen Frailty Indicator (GFI) criteria, which covers multiple dimensions, including physical, functional, psychological and social aspects and ranges from 0 to 15: a score of 4 or higher indicates frailty.*

2) Section “Discussion” 6^th^ paragraph contains the following addition:

*“Another possible limitation is that changes in clinical condition during hospitalization, e.g. recovering from a delirium, can have a different impact on handgrip strength and the m-30s-CST, due to different cognitive complexity.”*

Second comment: *Catabolic syndrome: what about parameters characterizing the “catabolic syndrome” in these patients? Please see and discuss: Curcio F et al. The reverse metabolic syndrome in the elderly: Is it a "catabolic" syndrome? Aging Clin Exp Res. 2018 Jun;30(6):547-554.*

We would like to thank the reviewer for this interesting theory of catabolic syndrome in these patients. It is certainly true that the patients in this study are in a catabolic state. They often suffer from inactivity, malnutrition, and inflammation due to the presence of infectious diseases and/or cardiopulmonary problems, both acute and chronic. Treatment of these conditions can influence the severity of catabolism and impact multiple metabolic processes, muscle strength, and performance. It is unknown whether the impact of catabolism differs in terms of its impact on changes in muscle strength in the arms and legs, measured by handgrip strength, respectively, and its relationship to changes in physical function measured with the m-30s-CST on the SPPB and Barthel ADL.

However, we believe the impact on muscle strength in the legs is greater than in the arms because these muscle groups are more inactive due to the hospitalization, and the impact on physical function measured by SPPB and Barthel ADL is also greater. Even with recovery through treatment of the acute illness (inflammation), malnutrition, and exercise intervention, the effect on leg strength could be greater than that on handgrip strength, as will the effect on physical function. We therefore believe that the catabolic syndrome supports our findings.

Changes/additions in the manuscript:

1) Section “Discussion” 6^th^ paragraph contains the following addition:

*“Furthermore changes in catabolic state due to treatment of the acute illness, e.g. with severe inflammation, malnutrition and immobility can impact changes in muscle strength (14). However, the extent to which catabolism differentially impacts changes in upper-limb (handgrip strength) versus lower-limb (m-30s-CST) muscle strength, and their respective relationships to physical function changes (SPPB and ADL-BI), remains unknown.”*

**Reviewer #2:**

This is a well-written and interesting paper. It provides practical insight into functional assessment in frail geriatric patients and highlights the limitations of handgrip testing in this setting. Some methodological limitations, however, should be considered:

First comment: The sample size is relatively small and derived from a single center, which limits generalizability.

We think the reviewer is making a valid point about generalizability of the results. Although we found a 95.6% level of certainty by performing a post-hoc analysis to assess the responsiveness of the m-30s-CST, study sample was relative small derived from a single center.

Changes/additions in the manuscript:

1) Section “Discussion” last paragraph contains the following addition:

*Furthermore a future multicenter study is needed to confirm findings before generalizing the results and including muscle mass in addition to muscle strength as an important characteristic of sarcopenia according to EWGSOP-2 and more functional outcomes like falls, hospital readmissions, recovery of autonomy, resilience, rehabilitation outcome and discharge planning.*

Second Comment: A large number of exclusions may have introduced selection bias. This also underlines a limitation in the applicability of the m-30s-CST in the acute hospital setting.

We believe the reviewer is making a valid point regarding potential selection bias.

Indeed only 92 patients of the 371 geriatric patients admitted in the hospital and 297 were excluded. However in 230 patients (78%) there was a logistical or ethical reason for exclusion, such as admission outside the geriatric ward, inability to enroll within 72 hours due to researcher availability, discharge within 72 hours preventing a second assessment, a palliative care plan agreed upon at admission or patients were wheelchair dependent prior to admission. For 57 patients, there were patient-related reasons, the majority of which (n=36) were not instructable due to severe cognitive problems. Of these patients however we have no data. Theoretically this could have introduced selection bias and have impact on the applicability of the m-30s-CST or HGS. However, the majority of the exclusions were due to logistic reasons and only in 21 patients very low physical performance was the reason for exclusion. For wheelchair-bound geriatric patients, measuring the HGS is feasible, while the m-30s-CST is not. The study was designed to investigate the responsiveness of muscle strength measured with the m-30s-CST or HGS to changes in physical function, as expressed in ADL-BI and SPPB, during hospitalization in geriatric patients who were independently mobile prior to hospital admission. Therefore we think the exclusion rate is not a limitation in the applicability of the m-30s-CST in the acute hospital setting in patients with a hospital independent mobility.

Changes/additions in the manuscript:

1) Section “Discussion”, 5^th^ paragraph contains the following addition:

*This study has several possible limitations that should be acknowledged. Of the 371 screened patients, only 92 were ultimately included in the study. In view of the high number of patients that are excluded, there may be selection bias. Because the main reasons for exclusion were logistical and ethical, we believe this does not substantially limit the applicability of the m-30s-CST in the acute hospital setting for geriatric patients who were independently mobile prior to hospitalization. In a small subset of geriatric patients who become (temporarily) wheelchair-bound due to acute illness, HGS may perform better. However, in the majority of patients, our results indicate that change in m-30s-CST performance is a better predictor of in-hospital physical recovery (ADL-BI and SPPB). We do not, however, have specific data on the responsiveness of HGS to changes in physical function during hospitalization among geriatric patients who are (temporarily) wheelchair-bound.*

Third Comment: It is not entirely clear which level of ADL function is considered at admission — the one assessed at the time of hospitalization (which may already be unrealistic and influenced by multiple confounding factors) or the premorbid status immediately before the acute event. In any case, the definition of "incident disability" (new-onset disability) related to hospitalization remains conceptually problematic for the study’s interpretation.

We thank the reviewer for the comment. We agree with the reviewer that it is unclear what the premorbid ADL level was as a result of possible chronic problems and what arose as a result of the acute illness that led to the hospital admission. In our study, in accordance with the Barthel Index instructions, we examined the 24 to 48 hours prior to admission as a standard benchmark and level of functioning and independence for patients (*Mahoney FI, Barthel DW. Functional evaluation: the Barthel Index. Maryland state medical journal. 1965 Feb;14:61-65*). This could, of course, mean that patients were already ill at that time and/or that there was already an impact on their level of ADL functioning (e.g. due to a chronic disease), as the reviewer rightly point out.

The impact on changes in ADL at this point can vary for different patients. However, we were unable to correct for this in our study, and therefore we do not know with certainty what ADL functioning was like before the illness. In our opinion however this seems irrelevant for studying the influence of changes in muscle strength, measured by handgrip strength and changes in leg muscle strength, measured with the modified 30-second chair stand test, upon admission and immediately before discharge.. O’Brien and coworkers compared ADL between two weeks before admission, at admission and hospital discharge or two weeks after, in 83 frail patients (age 81+8), The ADL was assessed across various domains, such as dressing, walking, bathing, eating, getting in and out of bed, and toileting. They found a decline in many of these tasks in the majority of patients. Approximately 45% of patients showed a decline at hospital admission in dressing, walking, showering, getting in and out of bed, and toileting. However, compared between admission and discharge or two weeks after discharge, they found improvement or stability in approximately 80% of patients in dressing, walking, showering, getting in and out of bed, and toileting. These authors conclude that most decline occurs in the period from pre-hospital admission to hospital admission, while an improvement occurs in the majority of patients between hospital admission and discharge or two weeks after that time (*O'Brien et al., 'Impact of Hospitalization on Patients' Ability to Perform Basic Activities of Daily Living’. Canadian Geriatric Journal, Volume 26, Issue 4, December 2023*)

Changes/additions in the manuscript:

1) Section “Discussion” 7^th^ paragraph contains the following addition:

*A third potential limitation is the uncertainty regarding the premorbid ADL levels, which may reflect chronic impairments, acute illness-related new-onset disability leading to hospitalization, or both, and their impact on changes in HGS and m-30s-CST. Although clinically relevant, this distinction does not affect our primary research question.*

Forth Comment: The Cox analysis included only a few covariates and did not adjust for potential confounders such as length of stay, immobilization, or rehabilitation interventions.

Thank you for pointing out this issue. In the last sentence of the method section we pointed out that our sample size was too limited to correct for all relevant potential confounders. Furthermore you suggest more relevant potential confounders e.g. length of stay, immobilization or rehabilitation interventions. We have only data of length of hospital stay and adding it in the model does not seem to influence 2 year mortality outcome in our population.

Changes/additions in the manuscript:

1) Section “Discussion” 8^th^ paragraph contains the following addition:

*Furthermore the predictive power of the m-30s-CST and HGS on 2-year mortality remains uncertain, as the study was not powered for this outcome and we cannot adjust for a number of clinically important confounders, such as the impact of delirium or treatment of the disease. These findings thus require confirmation in larger, adequately powered multicenter studies.*

2) Section “Discussion” last paragraph contains the following addition:

*Furthermore a future multicenter study is needed to confirm findings before generalizing the results and including muscle mass in addition to muscle strength as an important characteristic of sarcopenia according to EWGSOP-2 and more functional outcomes like falls, hospital readmissions, recovery of autonomy, resilience, rehabilitation outcome and discharge planning.*

Fifth comment: No measure of muscle mass was included, preventing an integrated analysis of structural sarcopenia.

Thank you for pointing out this issue. Unfortunately we have no data of muscle mass in this study. We agree that low muscle strength is hallmark of sarcopenia according the EWGSOP-2 criteria. Additional research that examines muscle mass and muscle quality in addition to muscle strength will provide better insight into the impact of sarcopenia in these patients.

Changes/additions in the manuscript related to this comment

1) Section “Discussion” last paragraph contains the following addition:

*Furthermore a future multicenter study is needed to confirm findings before generalizing the results and including muscle mass in addition to muscle strength as an important characteristic of sarcopenia according to EWGSOP-2 and more functional outcomes like falls, hospital readmissions, recovery of autonomy, resilience, rehabilitation outcome and discharge planning.*

Sixth comment: Mortality cut-offs for the m-30s-CST were defined empirically rather than validated. Given this, I recommend performing a ROC curve analysis to identify the optimal cut-off value and quantify the test’s discriminative power (AUC). This would strengthen the statistical validity of the conclusions and allow direct comparison with handgrip strength.

Thank you for pointing out this issue. As recommended by the reviewer, we additionally performed a ROC curve analysis. The ROC curve shows that the m-30s-CST has an area under the curve (AUC) of 0.609, (CI 95%: 0.488- 0.731) P= 0.071 and indicates a weak predictive value. The HGS measured with the Jamar, has an AUC= 0.573. CI 95%: 0.455-0.691) P=0.221. These findings suggests that the m-30s-CST seems to be a (poor) predictor of 2y-mortality and HGS does not seem to be a predictor of 2y-mortality.

We addressed this issue in the methods, results and discussion section with these additions

1) Section ‘Materials and methods’ subsection ‘*Statistics’, last paragraph*

*“Furthermore a Receiver Operating Characteristics curve (ROC) analysis to identify the optimal cut-off value and quantify the test’s discriminative power (AUC) of the m-30s-CST and HGS for 2-year mortality. Subsequently Cox proportional hazard ratio (HR) analysis was performed to determine which of these variables could best predict 2-year mortality. “*

2) Section ‘Results’ subsection “Muscle strength parameters and two-year mortality”*, third and fourth paragraph*

*“The ROC curve shows that the m-30s-CST has an area under the curve (AUC) of 0.609 (CI 95%: 0.488- 0.731) with P= 0.071, showing a trend towards predicting two-year mortality. The ROC curve shows that the HGS has an AUC= 0.573 (CI 95%: 0.455-0.691) with P=0.221, showing no predictive value of two-year mortality.*

*(…)*

*HGS categorized as low (n=66; mean 13.0+6.0 kg) versus normal (n=26; mean 25.3+9.5 kg) according the EWGSOP-2 ( female <16kg and men < 27 kg) did not predict mortality (HR 0.969; CI-95%: 0.495-1.900; P=0.928) and patients with less than 6 (n=59; mean 2.0+1.8) compared with more 5 (n=33; mean 8.6+2.5) repetitions on the m-30s-CST had a significant higher mortality risk ( HR 2.739; CI-95%: 1.173-6.396; P=0.020)”*

3) Section ‘Discussion’, first paragraph

*Furthermore, we demonstrated that only the m-30s-CST and not HGS was possibly a weak predictor of two-year mortality. HGS with the cut-off value according to the EWGSOP-2 has no predictive value for 2-year mortality, however the m-30s-CST with a cut-off value of <6 repetitions is a significant predictor of 2 year mortality.*

4) Section ‘Discussion’, 8^th^ paragraph

*Furthermore the predictive power of the m-30s-CST and HGS on 2-year mortality remains uncertain, as the study was not powered for this outcome and we cannot adjust for a number of clinically important confounders, such as the impact of delirium or treatment of the disease. These findings thus require confirmation in larger, adequately powered multicenter studies.*

Seventh Comment: Functional outcomes beyond mortality (e.g., falls, readmissions, recovery of autonomy) were not assessed, although these would be clinically meaningful in this population.

We agree with the reviewer that factors e.g., falls, readmissions, recovery of autonomy are clinically meaningful outcomes. However we have no data on these topics. In future research these topics should be addressed

Changes/additions in the manuscript related to this comment

1) Section “Discussion” last paragraph contains the following addition:

*“Furthermore a future multicenter study is needed to confirm findings before generalizing the results and including muscle mass in addition to muscle strength as an important characteristic of sarcopenia according to EWGSOP-2 and more functional outcomes like falls, hospital readmissions, recovery of autonomy, resilience, rehabilitation outcome and discharge planning.”*

Eighth comment: Finally, the discussion and conclusions could more clearly highlight the key clinical message and what this study adds to daily geriatric practice.

We addressed this issue in the discussion section by rewriting the discussion section and by adding a conclusion section with a clinical key message. The “Revised manuscript with Track Changes” clearly shows the rewritten discussion and conclusion.

**Journal Requirements:**

When submitting your revision, we need you to address these additional requirements.

1. Please ensure that your manuscript meets PLOS ONE's style requirements, including those for file naming.

We adapted the manuscript now meeting the PLOS ONE style requirements.

2. We note that your Data Availability Statement is currently as follows: “All relevant data are within the manuscript and its Supporting Information files.” Please confirm at this time whether or not your submission contains all raw data required to replicate the results of your study. Authors must share the “minimal data set” for their submission

In the informed consent form, we stated that no data would be shared. Therefore, we can only provide specific data upon explicit request. We are, of course, willing to share this data if it does not conflict with the informed consent.

3. We note that you have included the phrase “data not shown” in your manuscript. Unfortunately, this does not meet our data sharing requirements. PLOS does not permit references to inaccessible data.

We have made some adjustments and rewritten the discussion section and removed this part in accordance with PLOS One requirements.

4. Your ethics statement should only appear in the Methods section of your manuscript. If your ethics statement is written in any section besides the Methods, please delete it from any other section.

We have adjusted this in the materials and methods section according to PLOS One requirements

5. Please include a separate caption for each figure in your manuscript.

We have adjusted this in the manuscript according to PLOS One requirements

6. Please upload separately your supporting tables with the file type 'Supporting Information'. Please ensure that each Supporting Information file has a legend listed in the manuscript after the references list. Please see our Supporting Information guidelines for more information: http://journals.plos.org/plosone/s/supporting-information.

We have adjusted this according to PLOS One requirements

We have a question regarding authorship. The first author is also the last author. This is because Sipers is both the author of the study and led the entire research, including the manuscript and most of the article. How does PLOS One handle this?

We hoped the reply letter has answered the questions and remarks to your satisfaction.

Sincerely,

Walther Sipers and co-authors
